# Do Pre‐Treatment Biopsy Characteristics Predict Early Tumour Progression in Feline Diffuse Large B Cell Nasal Lymphoma Treated With Radiotherapy?

**DOI:** 10.1111/vco.13032

**Published:** 2024-12-04

**Authors:** Valerie J. Poirier, Valeria Meier, Michelle Turek, Neil Christensen, Jacqueline Bowal, Matthew D. Ponzini, Stefan M. Keller

**Affiliations:** ^1^ Department of Clinical Studies, Ontario Veterinary College University of Guelph Guelph Ontario Canada; ^2^ Clinic for Radiation Oncology & Medical Oncology Vetsuisse Faculty Zurich, University of Zurich Zurich Switzerland; ^3^ Department of Surgical Sciences, School of Veterinary Medicine University of Wisconsin‐Madison Madison Wisconsin USA; ^4^ Small Animal Specialty Hospital North Ryde New South Wales Australia; ^5^ Department of Public Health Sciences, School of Medicine University of California Davis California USA; ^6^ Department of Pathology, Microbiology and Immunology, School of Veterinary Medicine University of California‐Davis Davis California USA

**Keywords:** cats, IMRT, KI‐67, lymphoma, treatment failure, tumour‐infiltrating lymphocytes

## Abstract

The standard of care treatment for localised feline nasal lymphoma (FeNL) is radiation therapy (RT). Early local or systemic failure occurs in 17%–45% of cats treated with RT with or without chemotherapy. The aim of this study was to determine if pre‐treatment biopsy characteristics could predict early tumour progression in FeNL. Inclusion criteria consisted of histologically confirmed FeNL, available paraffin blocks of diagnostic quality, localised to the sinonasal cavity on staging pre‐RT, treated with IMRT/IGRT (10 × 4.2 Gy) without chemotherapy and at least 1 year follow‐up. All pre‐RT biopsies were reviewed and evaluated with CD3, CD20, CD79a, pan‐CK and Ki‐67 immunohistochemistry and the mitotic activity index was determined. The primary endpoint was progression‐free survival (PFS) at 1 year and hazard‐ratios (HR) with confidence interval (CI) were calculated. Twenty‐eight cats fit the inclusion criteria, and all had diffuse large B‐cell lymphoma. Seventeen cats (61%) were progression free at 1 year. Of the 11 cats that progressed in the first year, two had local progression, two had both local and systemic progression and seven had systemic progression. The mitotic index (HR: 1.03, CI 0.9–1.19, *p* = 0.645), Ki‐67 (HR: 1.00, CI 0.98–1.02, *p* = 0.845) and > 30% of tumour‐infiltrating T cells (HR: 0.38, CI 0.09–1.56, *p* = 0.175) were not significantly associated with PFS. In this uniformly RT treated population of FeNL, none of the evaluated pre‐RT histologic parameters could predict early treatment failure.

## 1 Introduction

Lymphoma is one of the most common malignant tumours in cats and prognosis varies according to lymphoma subtype, anatomical location and staging [1]. Feline nasal lymphoma (FeNL) is the most common type of extra‐nodal lymphoma in cats [2]. At diagnosis, approximately 80% of FeNLs are localised to the nasal cavity without evidence of systemic disease, but systemic spread is ultimately observed in 17%–50% of cases [4, 5, 6, 7, 8, 9]. If untreated, cats with FeNL succumb to complications of local invasion or systemic disease.

The subtyping of FeNLs has been inconsistent because of different classification schemes applied, the extent to which tumours were characterised immunohistochemically and the type of sample available. FeNLs are most commonly high‐grade B‐cell lymphomas and in one study that classified tumours according to WHO criteria, diffuse large B‐cell lymphomas (DLBCL) dominated [3, 10, 11, 12, 13, 14]. The only exception is one study of 115 FeNL that found only 39% B‐cell lymphomas, 47% T cell lymphomas and 13% double positive lymphomas [15].

Ki‐67 is a nuclear protein involved in the regulation of cell proliferation, and its expression has been widely used to evaluate the proliferative activity of neoplastic cells in human and veterinary medicine [16, 17, 18, 19, 20, 21, 22, 23, 24, 25, 26]. In a previous study assessing 17 cases of FeNL treated with radiation therapy (RT), Ki‐67 was found to be prognostic for the response to RT and survival [7].

RT is the standard of care, however, survival rates vary widely [4, 5, 6, 7, 8, 9]. Approximately 17%–45% of cats with nasal lymphoma treated with RT alone will have systemic or local progression within the first year while approximately 50% will enjoy long‐term (> 2 years) remission [4, 5]. These data demonstrate that (1) cats respond differently to RT with early treatment failure or long‐term tumour control as main outcomes, (2) over 50% of cats with FeNL do not require adjunctive therapy if treated with RT alone because they do not develop the systemic disease, and (3) RT alone may not be the optimal treatment regimen for a subset of cats.

If one were able to distinguish cats with early treatment failure from cats with long‐term tumour control at the time of diagnosis, then alternate treatment protocols such as adjunctive chemotherapy could be considered for the former group. Unfortunately, current modes of prognostication are insufficient to capture this dichotomy of biological behaviours, which represents a major obstacle for patient care.

The aim of this study was to determine if pre‐treatment biopsy characteristics can predict disease progression in FeNL.

## 2 Methods

### 2.1 Case Selection and Sample Size Calculation

Forty‐five cats treated for a localised FeNL at one of 4 veterinary radiation oncology centres (University of Guelph, Canada (*n* = 15); University of Wisconsin‐Madison, USA (*n* = 10); University of Zurich, Switzerland (*n* = 18); Small Animal Specialty Hospital, Australia (*n* = 1)) were previously reported and prospectively reviewed for inclusion [27]. Inclusion criteria consisted of histologically confirmed FeNL, treated with a uniform 10 daily fractions of 4.2 Gy radiation protocol using intensity‐modulated radiation therapy/image‐guided radiation therapy (IMRT/IGRT). Lymphoma had to be localised to the nasal cavity at the time of diagnosis according to the staging tests performed. Minimum staging was head CT, thoracic imaging (radiographs or CT), abdominal imaging (ultrasound or CT) and cytology of regional lymph nodes. Minimum follow‐up time was 1 year from the first RT and histologic paraffin blocks had to be available for review. Cats that had evidence of systemic lymphoma and/or spread to regional lymph node and cats that received adjuvant chemotherapy (prior to, during or after RT before progression) were excluded. Information extracted from the medical records were age, weight, breed, sex, clinical signs, imaging performed and results, Adams stage [28], tissue sampling for staging and results, tumour volumes [gross tumour volume (GTV), clinical target volume (CTV), planning target volume (PTV)] at start of RT, if prednisone was used pre‐RT (yes or no), time to progression as well as progression location. The progression location was defined as ‘local’ if the tumour was confined to the nasal cavity, as ‘lymph node’ if the tumour had spread to a lymph node, or as ‘systemic’ if the tumour had progressed beyond the nasal cavity and lymph node. An initial sample size calculation was performed using an R language‐based ROC sample calculator (version 1.3.1) [29]. Assuming a type‐1 error of 0.05, a power of 0.8, an area under the ROC curve (AUROC) of 0.8 (a lower AUROC might not be clinically significant) and an allocation ratio of 3 (based on 1/4 of cats having early failure), the minimum sample size required was 28 cats.

### 2.2 Immunohistochemistry, Ki‐67, Mitotic Index and Tumour Infiltrating Lymphocytes

Seven consecutive, unstained sections mounted on positively charged slides were cut at the contributing institutions and shipped to the Leukocyte Antigen Biology Laboratory at the University of California, Davis (UCD), USA. Deparaffinisation of histological slides was carried out by immersing the slides in a series of xylene followed by decreasing concentrations of ethanol. Antigen retrieval was carried out using a citrate buffer (Agilent Dako, Target Retrieval Solution, Citrate pH 6.1 (10 ×), #S1699) and a pressure cooker on ‘high’ setting for 5 min, followed by natural depressurization for 30 min and a cool down period at room temperature for 20 min. Blocking was done by applying 10% normal serum in PBS for 15 min at room temperature using serum corresponding to the species the secondary antibody was raised in. The following immunohistochemical markers and concentrations were used for immunohistochemistry: monoclonal rat anti‐canine CD3 epsilon (clone CD3‐12, IgG1, Dr. PF Moore, Davis, dilution 1:10), polyclonal rabbit anti‐human CD20 (Epredia, #RB‐9013‐R7, dilution 1:200), monoclonal mouse anti‐human CD79A (HM57, IgG1, Santa Cruz Biotechnology, #sc‐53 208, dilution 1:200), monoclonal mouse anti‐human Cytokeratin 1 (4D12B3, IgG1, Santa Cruz Biotechnology, #sc‐65 999, dilution 1:250), monoclonal mouse anti‐human Ki‐67 (MIB‐1, IgG1, Santa Cruz Biotechnology, #sc‐101 861, dilution 1:300). Slides were incubated with the primary antibody in a moist chamber for 60 min at room temperature followed by three washes with phosphate‐buffered saline (PBS). Slides were then drained and incubated with the ImmPRESS HRP Polymer Detection Kit for 30 min at room temperature (Vector Laboratories, #MP‐7402 for mouse, #MP‐7404 for rat and #MP‐7401 for rabbit) and rinsed with PBS three times. Subsequently, the NovaRED Substrate (Vector Laboratories, #SK‐4800) was applied followed by a rinse in distilled water and counterstaining for 5 s using Mayer's Haematoxylin. Two B‐cell markers (CD79 and CD20) were performed as redundancy because CD79 does not work reliably in all cats. A pan‐CK was performed to rule out nasal carcinoma. All slides were reviewed by a board‐certified pathologist with an expertise in lymphoproliferative diseases (SMK). Lymphocyte lineage was assigned as follows: T cell: CD3+, CD20−, CD79−; B cell: CD3−, CD20+ and/or CD79+. Intranuclear reactivity of CD79a was considered unspecific and was disregarded. Because 10 high power fields were not available in all cases due to small sample size, the mitotic activity was assessed as mitotic activity index (MAI), that is, the number of mitotic figures across all high‐power fields (HPFs) divided by the number of HPFs (one HPF = 0.237 mm^2^) [30]. Ki‐67 positivity was estimated as percent positive neoplastic cells in the most proliferative part of the tumour. Intratumoral T‐cells were categorised semi quantitatively as <= 5%, 6%–30% and > 30% of all lymphocytes. All neoplasms were classified based on the Histological Classification of Haematopoietic Tumours of Domestic Animals [31].

### 2.3 Post Hoc Analysis

To keep a uniform population and because our sample size was large enough, we elected to only include cats with diffuse large B‐cell lymphoma (DLBCL) for analysis and excluded cats without DLBCL on histological review post‐IHC.

### 2.4 Statistical Analysis

For continuous variables, descriptive statistics were reported as appropriate with median and range, mean and standard deviation (sd). For the analysis of mitotic activity, the MAI was converted into mitotic count, that is, the equivalent number of mitoses per 10 HPFs. For the percentage of Ki‐67 positive cells, the upper end of the observed range was used. ‘Progression‐free’ survival was defined as the interval from the first RT until systemic/local progression or death was observed due to any cause. The cut‐off for early treatment failure was set at 1 year and was chosen based on previously published studies that demonstrated a markedly decreased mortality rate after 1 year. Cats were censored if they were alive without progression at the time of the analysis. Kaplan–Meier curves were fit for progression‐free survival by categorical variables. Cox proportional hazards models were fit to assess Ki‐67, mitotic activity index and reactive T‐cells with progression‐free survival. Statistical analysis was conducted using R version 4.2.1 [32]. A *p* value of < 0.05 was considered significant.

### 2.5 Cell Line Validation Statement

No cell lines were used.

## 3 Results

### 3.1 Cat Characteristics

**FIGURE 1:**
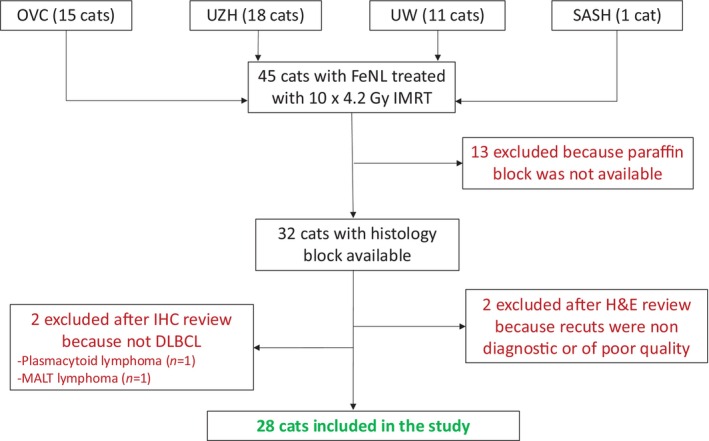
Cat selection algorithm. DLBCL, Diffuse large B‐cell lymphoma; FeNL, Feline nasal lymphoma; H&E, Haematoxylin and eosin; IHC, Immunohistochemistry; IMRT, Intensity‐modulated radiation therapy; MALT, Mucosa‐associated lymphoid tissue; OVC, Ontario Veterinary College; SASH, Small Animal Specialty Hospital; UW, University of Wisconsin; UZH, Vetsuisse Faculty Zurich.

Out of 45 reviewed cases, 32 cats had paraffin blocks available and underwent histology review (Figure 1). After histology review, tissues from 30 cats were of sufficient quality for immunohistochemistry and of these, 28 cats fit the inclusion criteria and were DLBCLs. The cat characteristics are described in Table 1. All cats received 42 Gy in 10 daily fractions. Radiation therapy with IMRT and IGRT was performed by a board‐certified radiation oncologist and according to the respective institutions' guidelines which could be variable but included a gross tumour volume (GTV) that was tumour on CT scan, clinical target volume (CTV) expansion minimum 1 cm in the nasal cavity but often included whole nasal cavity and nasopharynx and planned target value (PTV) expansion at a minimum of 2 mm. PTV dose coverage and organ at risk (OAR) dose were chosen based on the attending radiation oncologist. The radiation dose details are reported in Table S1.

**TABLE 1:** Cat characteristics.

Variables	*N* = 28 (100%)
Age (years)	Median: 10.3 (range: 3.5–14.4)
Sex	Female spayed: *N* = 16 (57%)
	Male neutered: *N* = 12 (43%)
Breed	Domestic short hair (*N* = 16)
	Maine coon (*N* = 3)
	Russian blue (*N* = 2)
	One of each: Abyssinian, Balinese, Bengal, Bombay, Burmilla, Siamese, Singapura
Weight (kSg)	Median: 4.7 (range: 2.4–7.3)
Staging	
Thorax radiographs	*N* = 5 (18%)
CT thorax	*N* = 23 (82%)
Abdominal ultrasound	*N* = 24 (86%)
CT abdomen	*N* = 4 (14%)
Cytology lymph node	Yes: *N* = 27 (96%), No: *N* = 1 (4%)
	Mandibular lymph nodes: *N* = 25 (89%)
	Medial retropharyngeal lymph nodes: *N* = 22 (79%)
Cytology liver	Yes: N = 18 (64%), No: *N* = 10 (36%)
Cytology spleen	Yes: *N* = 19 (68%), No: *N* = 9 (32%)
Modified Adams stage [28]	1: *N* = 4 (14%)
	2: *N* = 2 (7%)
	3: *N* = 13 (46%)
	4: *N* = 9 (32%)/(4a (8), 4b (1))
Steroids use	Yes: *N* = 24 (86%), No: *N* = 4 (14%)
GTV volume (cm^3^)	Median: 8.9 (range: 0.1–36.2)
CTV volume (cm^3^)	Median: 20.2 (range: 3.1–52.3)
PTV volume (cm^3^)	Median: 34.3 (range:7–74.8)

Abbreviations: CT, computed tomography; CTV, Clinical target volume; GTV, Gross tumour volume; PTV, Planned target volume.

### 3.2 Histological Evaluation and Immunohistochemistry

**FIGURE 2:**
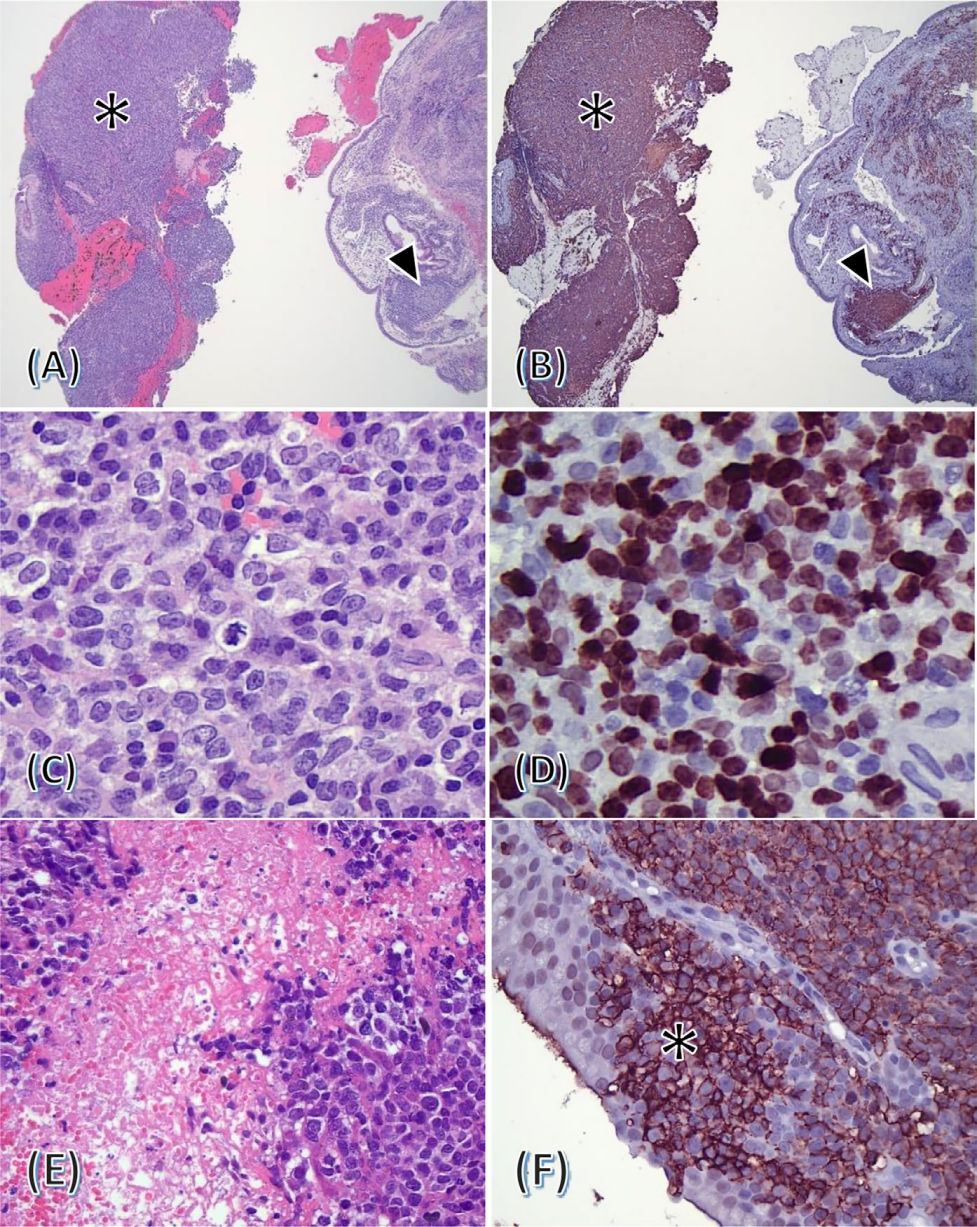
Histological and immunohistochemical characteristics of feline nasal lymphoma. (A) and (B) Low‐power appearance of diffuse large B‐cell lymphoma (asterisk) and normal mucosa with reactive lymphoid follicle (arrowhead). (A) haematoxylin and eosin, 4 × objective. (B) CD20 IHC with haematoxylin counterstain, 4 × objective. (C) High‐power magnification immunoblastic cells and atypical mitotic figure, haematoxylin and eosin, 40 × objective. (D) 80%–90% of lymphocytes are positive for Ki‐67, Ki‐67 IHC with haematoxylin counterstain, 40 × objective. (E) Areas of necrosis, haematoxylin counterstain, 20 × objective. (F) Epitheliotropic B cells (asterisk) invading the overlying epithelium, CD20 IHC with haematoxylin counterstain, 20 × objective.

Out of 32 cases, 2 cases could not be classified definitively because of insufficient tissue quality and/or quantity and 2 cases were re‐classified as suspect mucosa‐associated lymphoid tissue (MALT) lymphoma and suspect plasmacytoid lymphoma, respectively (Figure 1). Clonality testing to substantiate the diagnosis of MALT lymphoma failed due to insufficient tissue remaining. Further immunohistochemical workup to characterise the suspect plasmacytoid lymphoma was not possible due to insufficient tissue remaining. All cases displayed strong reactivity for CD20 and weak to moderate reactivity for CD79. One case was additionally positive for CD3, and no case displayed reactivity for cytokeratin (Figure 2).

### 3.3 Progression Free Survival and Outcome

**FIGURE 3:**
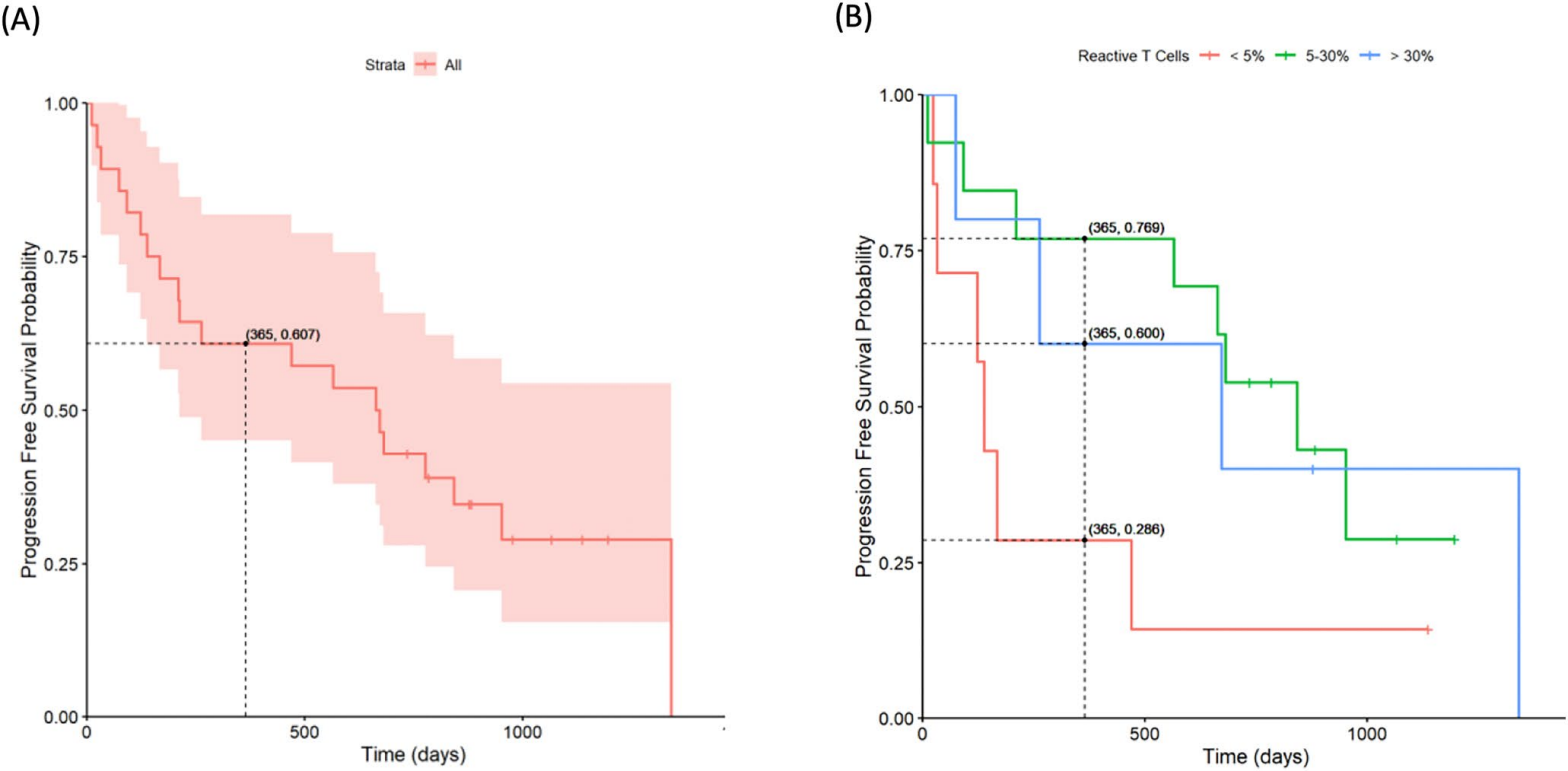
Progression‐free survival with a 95% confidence interval for all cats (A) without stratification and (B) stratified by the percentage of tumour‐infiltrating T cells. Tick marks represent censored patients.

For the remaining 28 cats, the median progression‐free survival was 668 days. Eight cats were alive without progression and were censored (median: 681 days, range: 367–913 days). Seventeen cats (61%) were progression‐free at 1 year and 11 (39%) had progression in the first year (median: 113 days, range: 12–263 days) (Figure 3a). Of the 11 cats that progressed in the first year, two had local progression, two had both local and systemic progression and seven had systemic progression.

### 3.4 Mitotic Activity Index

The mean mitotic index of 23 cats was 5.0 (SD: 3.8, range: 1–15). The mitotic activity index could not be performed in 5 cats because of insufficient tissue. The mitotic index for cats that progressed in the first year (mean: 5.3 (SD: 3.5, range: 1–13)) was not significantly different from the mitotic index of the cats that did not progress (mean: 4.5 (SD: 4.2 range: 1–15)), with a hazard ratio of 1.03 (95% CI: 0.9–1.19) *p* = 0.645.

### 3.5 Ki‐67

The mean Ki‐67 for all cats was 68.5% (SD: 23.1%, range: 10%–98%). The Ki‐67 for cats that progressed in the first year (mean: 67.2% (SD: 20.7%, range: 40%–95%)) was not significantly different from the Ki‐67 of the cats that did not progress (mean: 70.3% (SD: 26.8%, range: 10%–98%)), with a hazard ratio of 1.00 (95% CI: 0.98–1.02) *p* = 0.845.

### 3.6 Tumour‐Infiltrating Lymphocytes

Out of 25 cats for which reactive T‐cells could be determined, 7 (28%) had ≤ 5% of reactive T cells, 13 (52%) had 6%–30% of reactive T‐cells and 5 (20%) had > 30% of reactive T cells. The percentage of reactive T‐cells was not significantly associated with progression‐free survival. Hazard ratio (6%–30%): 0.36 (95% CI: 0.12–1.07), *p* = 0.066, hazard ratio (> 30%): 0.38 (95% CI: 0.09–1.56), *p* = 0.179. (Figure 3b).

## 4 Discussion

This study aimed to identify pre‐treatment biopsy characteristics that could predict early tumour progression in DLBCL FeNL treated with radiotherapy. While prognostication of FeNL based on biopsy characteristics has been attempted before, this is the first study that is based on a homogeneous group of lymphoma subtypes (DLBCL) and a standardised radiotherapy protocol. We found that neither mitotic activity index, Ki‐67 proliferation or tumour infiltrating lymphocytes were predictive of outcome.

In other species and tumour types, histological and immunohistochemical characterisation of tumour biopsies is an integral part of prognostication. In canine high‐grade multicentric lymphoma treated with standard chemotherapy alone, immunophenotyping is a known prognostic factor as dogs with B‐cell lymphoma live twice as long as dogs with T‐cell lymphoma [1, 33]. While the majority of FeNLs are B‐cell lymphomas, the relationship between immunophenotype, grade and outcome after RT has not been investigated [3, 11, 12, 13, 14]. Ki‐67 is a proliferation marker that is routinely performed by veterinary diagnostic laboratories on formalin fixed and paraffin embedded tissue specimen. The percentage of Ki‐67 positive cells is a prognostic factor in human lymphoma and multiple feline tumours [17, 18, 19, 20, 26]. In canine lymphoma, Ki‐67 has been demonstrated to predict grade [21, 22] and outcome in high‐grade B‐cell lymphoma treated with chemotherapy [23, 24].

The outcome of cats included in our study is similar to what was reported previously for cats treated with RT [4, 5]. Treatment failure prior to 1 year occurred in 40% of the cats in this study. Unfortunately, none of the histological parameters assessed could predict early treatment failure. Both the mitotic activity index and Ki‐67 are a measure of proliferation and were equivalent in both groups. This is in contrast with the only published study examining the relationship between Ki‐67, response to RT and outcome in cats with FeNL [7]. In this study, cats with a Ki‐67 index of more than 40% had a better outcome (median survival of 582 days) compared to cats with a Ki‐67 index of less than 40% (median survival of 77 days) [7]. However, the sample size for the previous study was only 17 cats with localised lymphoma (9 and 8 cats per group) treated with RT only using orthovoltage, a rather superficial and outdated RT technique [7]. The mean Ki‐67 index for our cat population was higher (68.5%) and only 4 cats had Ki‐67 under the 40% cut‐off of the previous study with the very variable outcome (3 cats being still alive without progression at 735, 735 and 742 days and 1 cat developing systemic lymphoma at day 132). It is possible that the population of cats was different as all cats in our study population had B‐cell high‐grade lymphoma which is the most common type of lymphoma in the sinonasal cavity of cats. We could not extrapolate our results to other immunophenotypes and the immunophenotype was not evaluated in the previous Ki‐67 study [7].

In cats, tumour‐infiltrating lymphocytes (TILs) have been investigated in the context of mammary and melanocytic tumours. TILs are defined as lymphocytes within and around cancer cells and have been associated with a survival benefit in some human cancers [34, 35]. In feline mammary carcinomas, increased proportions of CD8+ TILs were linked to extended periods of disease‐free survival and overall survival, while higher proportions of intratumoral CD4+ TILs were associated with positive lymph node status [36]. In feline melanocytic tumours, TILs were associated with histologic features of malignancy and loss of melanocytic‐specific markers [36]. In humans with DLBCL, patients displaying a high proportion of TILs have a significantly better prognosis than patients with a low proportion of TILs [37, 38, 39]. The fact that the percentage of reactive T cells was not significantly associated with progression‐free survival in our study could be due to several reasons such as the small number of cats in each category, differences in study design, treatment regimen or disease biology between human and feline DLBCL. In addition, neoplastic lymphocytes and TILs were sometimes in close proximity or contiguous with mucosa‐associated lymphoid tissue (MALT). The juxtaposition of reactive and neoplastic T cells as well as the high variability of T cells in different parts of the tumour might have resulted in imprecise TIL estimates in some cases. Lastly, a confounding factor in the histological interpretation of biopsies in this study was the small sample size in some cases. It is possible that the assessed areas were not representative of the most aggressive areas of the tumour or that the total tissue surface area available for quantification was insufficient for a robust statistical analysis.

In conclusion, we did not identify a pre‐treatment biopsy parameter that could predict early failure in cats with DLBCL treated with radiation therapy. Future studies should be directed at increasing cohort size, assessing other lymphoma subtypes, and utilising automated image analysis for more precise and reproducible quantification of mitotic figures and immunohistochemical markers. Furthermore, a more comprehensive interrogation of TILs that considers spatial distribution and clonality might be helpful in determining the prognostic value of this feature. Such advancements will be critical in refining treatment protocols and improving outcomes for feline patients with FeNL.

## Conflicts of Interest

The authors declare no conflicts of interest.

## Supporting information


**Table S1.** Radiation dose characteristics.

## Data Availability

All relevant data are included in the manuscript.
